# Inhibition of platelet activation suppresses reactive enteric glia and mitigates intestinal barrier dysfunction during sepsis

**DOI:** 10.1186/s10020-022-00562-w

**Published:** 2022-11-18

**Authors:** Bo Cheng, Mengyu Du, Shuxuan He, Lan Yang, Xi Wang, Hui Gao, Haiqing Chang, Wei Gao, Yan Li, Qiang Wang, Yansong Li

**Affiliations:** 1 Department of Anesthesiology & Center for Brain Science, The First Affiliated Hospital of Xi’an Jiaotong University, Xi’an, 710061 Shaanxi China; 2 Department of Anesthesiology, The First Affiliated Hospital of Wenzhou Medical University, Wenzhou, 325000 Zhejiang China

**Keywords:** Sepsis, Intestinal barrier dysfunction, CD40L-CD40, Enteric glia cells, *S*-Nitrosoglutathione

## Abstract

**Background:**

Intestinal barrier dysfunction, which is associated with reactive enteric glia cells (EGCs), is not only a result of early sepsis but also a cause of multiple organ dysfunction syndrome. Inhibition of platelet activation has been proposed as a potential treatment for septic patients because of its efficacy in ameliorating the organ damage and barrier dysfunction. During platelet activation, CD40L is translocated from α granules to the platelet surface, serving as a biomarker of platelet activation a reliable predictor of sepsis prognosis. Given that more than 95% of the circulating CD40L originate from activated platelets, the present study aimed to investigate if inhibiting platelet activation mitigates intestinal barrier dysfunction is associated with suppressing reactive EGCs and its underlying mechanism.

**Methods:**

Cecal ligation and puncture (CLP) was performed to establish the sepsis model. 24 h after CLP, the proportion of activated platelets, the level of sCD40L, the expression of tight-junction proteins, the intestinal barrier function and histological damage of septic mice were analyzed. In vitro, primary cultured EGCs were stimulated by CD40L and LPS for 24 h and EGCs-conditioned medium were collected for Caco-2 cells treatment. The expression of tight-junction proteins and transepithelial electrical resistance of Caco-2 cell were evaluated.

**Results:**

In vivo, inhibiting platelet activation with cilostazol mitigated the intestinal barrier dysfunction, increased the expression of ZO-1 and occludin and improved the survival rate of septic mice. The efficacy was associated with reduced CD40L^+^ platelets proportion, decreased sCD40L concentration, and suppressed the activation of EGCs. Comparable results were observed upon treatment with compound 6877002, a blocker of CD40L-CD40-TRAF6 signaling pathway. Also, *S*-nitrosoglutathione supplement reduced intestinal damage both in vivo and in vitro. In addition, CD40L increased release of TNF-α and IL-1β while suppressed the release of *S*-nitrosoglutathione from EGCs. These EGCs-conditioned medium reduced the expression of ZO-1 and occludin on Caco-2 cells and their transepithelial electrical resistance, which could be reversed by CD40-siRNA and TRAF6-siRNA transfection on EGCs.

**Conclusions:**

The inhibition of platelet activation is related to the suppression of CD40L-CD40-TRAF6 signaling pathway and the reduction of EGCs activation, which promotes intestinal barrier function and survival in sepsis mice. These results might provide a potential therapeutic strategy and a promising target for sepsis.

**Supplementary Information:**

The online version contains supplementary material available at 10.1186/s10020-022-00562-w.

## Background

Sepsis is a major health concern for intensive care unit (ICU) patients worldwide and is associated with high mortality rates in all countries (Vincent et al. 2014). During the ICU stay, 29.5% of patients developed sepsis, of which 25.8% died of multiple organ dysfunction syndrome (MODS) (Vincent et al. 2014). It is believed that the failure of the intestinal barrier with elevated bacterial translocation is a crucial driving force for MODS during sepsis (Zhou and Verne 2018), and early improvement of intestinal barrier function may be the key to prevent sepsis from endotoxemia to MODS (Armacki et al. 2018).

Composed of physical, chemical, biological and immune barriers (Baumgart and Dignass 2002), the intestinal barrier is the first line of defense against antigens, bacterial toxins, and pathogens in the hostile intestinal environment (Blikslager et al. 2007). Adjacent intestinal epithelial cells form the continuous physical barrier through tight junctions to regulate the intestinal permeability (Peterson and Artis 2014). Disruption of intestinal epithelial tight junctions may cause hyperpermeability which can be observed in both septic animals (Dominguez et al. 2013) and patients (Klaus et al. 2013). In the early stage of sepsis, the intercellular spaces among intestinal epithelial cells were expanded and the expression of tight junction proteins such as claudin-2, claudin-4, occludin and ZO-1 were decreased (Obermuller et al. 2020; Otani et al. 2020). Then, the apoptosis and damage of intestinal epithelial cells induced intestinal barrier dysfunction, which progressed rapidly to severe sepsis (Hu et al. 2019). Thus, mitigating intestinal barrier dysfunction may be a potential strategy for the treatment of sepsis.

The Enteric Nervous System is the second largest nerve system, composed of enteric neurons and enteric glial cells (EGCs) that share many characteristics with astrocytes (Jessen and Mirsky 2005). Although both enteric neurons and EGCs are associated with intestinal epithelial cells, accumulating evidence suggests that EGCs are supportive cells for neurons and play a key role in the regulation of epithelial homeostasis, matrix remodeling, immunity, and inflammation without neuronal activity (Schneider et al. 2019). Moreover, EGCs widely reside throughout the submucosal plexuses of intestine tract (Gulbransen and Sharkey 2012), an ideal position to interact with intestinal epithelial cells and maintain their tight junction by secreting *S*-nitrosoglutathione (GSNO) directly (Neunlist et al. 2014; Savidge et al. 2007). When encountering bacterial or inflammatory challenges, EGCs were activated, which in turn secreted TNF-α (Chelakkot et al. 2018), IL-1β (Rosenbaum et al. 2016) and other chemokines/cytokines (Van Landeghem et al. 2009) that disassociated the tight junction proteins and induced intestinal barrier hyperpermeability (Grubisic and Gulbransen 2017).

In septic patients, platelets were activated and sequestrated in the intestine, which could be used to precede clinical signs of sepsis and predict the outcomes (Sigurdsson et al. 1992; Wang et al. 2018). Although septic patients can benefit from the inhibition of platelet activation (Dewitte et al. 2017), the underlying mechanism remains obscure. Clinical evidence has shown that the level of CD40 ligand (CD40L) in serum is higher in patients with sepsis (Liang et al. 2021; Lorente et al. 2017) and is associated with decreased survival rate (Lorente et al. 2011). During platelet activation, CD40L is translocated from α granules to the platelet surface, which is positively correlated with plasma CD40L. Indeed, plasma CD40L has been reported as a biomarker of platelet activation such as PAC-1 and CD62p, and serves as a reliable predictor of sepsis prognosis (Wegrzyn et al. 2021). It is estimated that more than 95% of the circulating CD40L originates from activated platelets (Andre et al. 2002), and can modulate adaptive immune responses (Ma et al. 2022). Since platelet-derived CD40L can activate glial cells to damage the blood–brain barrier in hypertension (Bhat et al. 2017), we speculated that the activated platelets induce the intestinal barrier dysfunction, which is associated with the upregulation of CD40L-CD40 pathway in reactive enteric glia during sepsis.

## Materials and methods

### Animals

All animal experiments in this study were approved by the Ethics Committee for Animal Experimentation of the Xi’an Jiaotong University (Xi’an, China, 2018-107). Male C57BL/6 mice (6–8 weeks old) were obtained from Experimental Animal Center of Xi’an Jiaotong University (Xi’an, China) and raised in standard cages under controlled conditions with a 12-h light/dark cycle at 21 ± 2 °C temperature and 60–70%.

### Sepsis model and drug administration

Cecal ligation and puncture (CLP) was performed to establish the sepsis model with some modifications as described previously (Rittirsch et al. 2009). Briefly, we anesthetized the mice with isoflurane inhalation and injected lidocaine subcutaneously local to incision as an analgesic before surgery. The cecum was exteriorized through a 1–2 cm longitudinal midline abdominal incision. Then 75% of the cecum was ligated and punctured twice with a 21-G needle to externalize feces. All mice were subcutaneously injected with 1 mL 0.9% normal saline for fluid resuscitation and administrated a single dose of antibiotics (ceftriaxone at 30 mg/kg and clindamycin at 25 mg/kg) immediately after CLP. Subsequently, mice were temporarily placed on a heating pad to maintain the body temperature until fully recovered from anesthesia. The sham-operated mice underwent the same procedure without ligation and puncture. In the additional groups of mice, cilostazol (10 mg/kg) was diluted from 0.5% carboxymethyl cellulose sodium salt (CMC) and administrated orally 2 h prior to and at 12 h after CLP to inhibit platelet activation (Chang 2015), compound 6877002 (10 μmol/kg) was injected intraperitoneally 2 h prior to and at 12 h after CLP to block CD40L-CD40 signaling pathway (Zarzycka et al. 2015) and GSNO was administrated intraperitoneally at 10 mg/kg/day for 7 days before CLP to verify its function on intestinal hyperpermeability (Savidge et al. 2007). 24 h after CLP, mice were sacrificed, and blood or tissue samples were collected for further experiments.

### Intestinal barrier permeability test

The intestinal barrier permeability was evaluated by fluorescein isothiocyanate (FITC)-dextran test 24 h after CLP. Briefly, overnight fasted mice were orally gavaged with 25 mg/mL of 4 kDa FITC-Dextran (0.5 mg/g body weight; Sigma-Aldrich). After 1.5 h, plasma was collected, and plasma fluorescence was measured at excitation and emission wavelengths of 485 nm and 535 nm, respectively.

### Water content

Intestinal tissues were excised and weighed immediately (wet weight) and dried at 60 °C for 48 h before weighing (dry weight). The water content represented the degree of intestinal edema and was calculated as follows: water content (%) = (wet weight − dry weight)/wet weight × 100%.

### Bacterial content

The mesenteric lymph nodes, lung and liver tissues were homogenized, and the supernatant was obtained by centrifugation. After serial dilutions, 500 μL of each dilution was evenly spread onto blood agar plates, incubated at 37 °C for 24 h, and the colony forming units (CFUs) were counted.

### Histological damage score analysis

The histological damage score was estimated after formalin fixation, paraffin embedding and hematoxylin/eosin staining of sections. The images were observed by a light microscope, and a representative field was chosen for assessment. The intestinal damage score was graded as follows: 0, normal mucosal villi; 1, minor subepithelial space and capillary congestion; 2, extensive subepithelialspace with little epithelial layer lifting from the lamina propria; 3, massive epithelial layer lifting from the lamina propria; 4, villi detachment and hemorrhage (Bi et al. 2020). The histological damage scores were recorded and analyzed by two investigators blinded to the experimental treatments. Grading of intestinal injury was measured as an average score. Statistical analysis of histological data was performed by an investigator blinded to the experimental treatments.

### Echocardiography

Echocardiography was performed 24 h after the CLP using Vevo 3100 with a 400 MHz probe (FUJIFILM SonoSite, lnc. JAPAN). The mice (prior removal of hair from the precardiac region) were anesthetized with isoflurane inhalation and the limbs were fixed. The long axis section of the left ventricle was evaluated, and the left ventricle movement was detected with M-mode echocardiography. The following four parameters were measured: left ventricular end diastolic diameter (LVEDD), left ventricular end systolic dimension (LVESD), left ventricular ejection fraction (LVEF) and left ventricular fractional shortening (LVFS). All data were processed under the same parameters and analyzed by investigator blinded to the experimental treatments. The value of each measurement index was the average value of three consecutive cardiac cycles.

### Complete blood counts

Whole blood samples were collected from the mice via eyeball enucleation in K2EDTA tubes for complete blood counts analysis. Complete blood counts, including platelets, white blood cells (WBC), neutrophil (NEUT), monocyte (MONO) and lymphocyte (LYMPH), were measured using an auto hematology analyzer (Mindray, BC-6800Plus, China).

### Flow cytometry

To analyze platelet surface CD40L, blood was collected in sodium citrate and stained with 1 μL of FITC anti-mouse CD41 Antibody (BioLegend) and 2.5 μL of PE anti-mouse CD154 (CD40L) Antibody (BioLegend) at room temperature in the dark, according to the manufacturer’s instructions. Then, the blood samples were incubated with a red blood cell lysis solution (Solarbio, China) and analyzed by flow cytometry (BD Biosciences, USA). Data analysis was performed using FlowJo (Ashland, OR). Scatter and staining with the FITC-anti-CD41 and PE-anti-CD40L antibodies were used to gate platelet populations. Cells were first gated by regions within a side scatter area (SSC-A) versus forward scatter area (FSC-A) plot, then by gating those populations in a SSC-A versus FITC-A plot. Activated platelets were defined as FITC-anti-CD41-A positive and PE-anti-CD40L-A positive (Additional file 1: Fig. S1).

### Soluble CD40L determination

Soluble CD40L (sCD40L) levels in serum was measured using ELISA kits (Cloud-Clone Corp, Wuhan, China) according to the manufacturer’s recommendations.

### Immunofluorescence staining

Intestinal tissues were collected and fixed overnight in 4% paraformaldehyde. The fixed intestinal tissues were OCT-embedded, snap-frozen, and prepared into 16 μm sections for immunofluorescence staining. Cell samples including the primary EGCs, primary astrocytes and Caco-2 cells were seeded in 24-well plates plated with cell climbing slices respectively. After stimulation, cell samples were fixed in 4% paraformaldehyde and washed thrice with PBS for immunofluorescence staining. After permeabilization and blocking, the tissue sections or cell samples were incubated with the respective primary antibodies overnight at 4 °C. Briefly, the primary antibodies consisted of chicken anti-GFAP (1:200, GeneTex, Irvine, CA, USA), rabbit anti-Iba-1 (1:200 Abcam, Cambridge, UK), rabbit anti-Sox10 (1:500, Abcam, Cambridge, UK), anti-CD40 (1:200, Abcam, Cambridge, UK), and ZO-1 (1:200, Abcam, Cambridge, UK) antibodies. Next, the tissue sections or cell samples were washed thrice with PBS and incubated with A488 anti-chicken (1:1000, GeneTex) or A594 anti-rabbit (1:1000, GeneTex) antibodies at 37 °C for 2 h. DAPI (1:1000, Invitrogen) was used for nuclei counterstaining. An Olympus FluoView FV1000 microscope (Olympus, Tokyo, Japan) was used to collect the fluorescent images of intestinal tissues and cell samples. Fluorescent intensity was analyzed by ImageJ.

### Western blot analysis

Protein was extracted from intestinal tissues and cells using RIPA buffer containing protease inhibitor cocktail and measured using a BCA protein assay kit. An equivalent of protein samples was separated by 12% SDS-PAGE and transferred to PVDF membranes for western blot analysis. After blocking with 5% skimmed milk for 1 h, the membranes were probed overnight at 4 °C with the following different primary antibodies: rabbit anti-GFAP (1:5000, GeneTex, Irvine, CA, USA), rabbit anti-CD40 (1:1000, Abcam, Cambridge, UK), rabbit anti-ZO-1 (1:1000, Affinity, USA), rabbit anti-occludin (1:5000, ABclonal, Wuhan, China), mouse anti-TRAF1 (1:100, Santa Cruz, CA), mouse anti-TRAF2 (1:100, Santa Cruz, CA), mouse anti-TRAF3 (1:100, Santa Cruz, CA), mouse anti-TRAF4 (1:100, Santa Cruz, CA), mouse anti-TRAF5 (1:100, Santa Cruz, CA), mouse anti-TRAF6 (1:100, Santa Cruz, CA), and rabbit anti-GAPDH (1:5000, ABclonal). Then, the membranes were incubated with secondary antibodies at room temperature for 1 h. The immunoreactive bands were detected using a chemiluminescence imaging system ChemiScope 6000 (Clinx, Shanghai, China), and the intensity was analyzed with ImageJ.

### Primary EGCs and astrocytes culture

As mentioned previously, primary EGCs were prepared from the intestinal tract of embryonic days 17–19 fetal mice (Li et al. 2021) and cultured in Dulbecco’s modified Eagle’s medium (DMEM) (Gibco Ltd., NY, USA) containing 10% fetal bovine serum (FBS), 1% glutamine (Sigma), and 1% penicillin and streptomycin solution (Pen Strep) (Gibco) at a density of 4 × 10^4^ cells/cm^2^. After 1 week, EGCs were isolated using 1 U/mL dispase (Roche, Basel, Switzerland) to eliminate the fibroblasts or epithelial cells and identified by GFAP and Sox 10 immunoreactivity.

As described previously, primary astrocytes were prepared from postnatal days 1–3 mice brains (Zhu et al. 2020) and cultured in DMEM (Gibco) containing 10% fetal bovine serum (FBS), 1% glutamine (Sigma), and 1% penicillin and streptomycin solution (Pen Strep) (Gibco) at a density of 4 × 10^4^ cells/cm^2^. After 1 week, the cells were shaken (200 rpm, 37 °C) overnight to eliminate nonspecific glia, such as microglia, and the astrocytes were identified by GFAP and Iba-1 immunoreactivity.

### Stimulation and siRNA transfection

To induce the cell sepsis model, EGCs were stimulated by single lipopolysaccharide (LPS) (10 μg/mL, *E. coli* O111:B4, Sigma-Aldrich, St. Louis, MO, USA), CD40L (1 μg/mL, PeproTech, Rocky Hill, NJ, USA) or LPS + CD40L. After 24 h, the cell culture supernatants were collected as EGCs-conditioned medium for cytokine measurement and Caco-2 cell treatment.

For the siRNA transfection, CD40-siRNA, TRAF6-siRNA, negative control siRNA (NC-siRNA), and siRNA-Mate transfection reagent were purchased from GenePharma Co. Ltd, (Shanghai, China). EGCs were transfected with CD40-siRNA, TRAF6-siRNA or NC-siRNA using siRNA-Mate according to the manufacturer’s protocols for 72 h before LPS and CD40L stimulation.

### Caco-2 culture and in vitro permeability assay

Caco-2 cells (CL-0050, Procell Life Science & Technology Co., Ltd., Wuhan, China) were cultured in DMEM (Gibco) containing 10% fetal bovine serum (FBS), 1% glutamine (Sigma), and 1% penicillin and streptomycin solution (Pen Strep) (Gibco). For in vitro permeability assay, Caco-2 cells were seeded at a density of 4 × 10^6^ cells/cm^2^ in 6.5-mm transwell dishes with 0.4 μm pore polycarbonate membrane inserts (3413; Corning). After 1 week of culture, transepithelial electrical resistance (TEER) was measured using an Electrical Resistance System (ERS) (Millipore) to confirm cell polarization and establish barrier characteristics (> 450 Ω/cm^2^). Then, Caco-2 cells were stimulated with EGCs-conditioned medium and GSNO (50 µM, Sigma) for 24 h before Western blot and immunofluorescence staining. Transepithelial electrical resistance was measured as Ω cm^2^ every hour after stimulation by subtracting the background resistance and multiplying with the monolayer surface area to calculate the relative value.

### Cytokine and GSNO measurements

TNF-α, IL-1β and GSNO were measured using ELISA kits as recommended by the manufacturer. Briefly, the supernatants were harvested from intestinal tissue homogenate, serum and EGCs-conditioned medium, and the impurity was removed by centrifugation at 12,000×*g* for 10 min. TNF-α (EMC102a.96) and IL-1β (EMC001b.96) ELISA kits were obtained from NeoBioscience (Shenzhen, China) to measure TNF-α and IL-1β production in vivo and in vitro. GSNO (ml08365974) ELISA kit was obtained from Mlbio (Shanghai, China) to measure GSNO production in vitro.

### Bioinformatics analysis

Bioinformatics analysis was performed using public databases. These data were downloaded from the GSE156905 dataset of the Gene Expression Omnibus (GEO) database. The single-cell sequencing data (GSE156905) were sourced from distal colon of adult mouse.

The “Read10×” function in the package was used to analyze single-cell sequencing data. Seurat objects were created using the “CreateSeuratObject” function. The “FilterCells” function was used to filter the data and remove the effects of dead cells and cell adhesion. The specific parameters used in the subsequent analysis are as follows: NFEATURE RNA > 200, nFEATURE RNA < 2500 and PERCENT.mt < 5, leaving 1384 cells and 17,047 genes. Data were normalized using the “NormalizeData” function with the following parameters: normalize. Method = “LogNormalize” and scale.factor = 1000. The “Find variable genes” function was used to calculate highly variable genes and set to default values. The “ScaleData” function was further used to normalize the data and remove the source of variation. The Uniform Manifold Approximation and Projection (UMAP), was obtained via analysis using the “BiocManager” packets in the R language.

Screening and annotation of cell marker genes in different subpopulations. The screening and annotations of different cell subpopulations were compared among all cell subpopulations and obtained genes per subgroup. The identification of each cell was based on the differential expression of characteristic genes among the various cell clusters. GFAP has long been a specific marker of astrocyte (Yang and Wang 2015), which shares many characteristics with EGCs (Gulbransen and Sharkey 2012; Jessen and Mirsky 1980; Yang and Wang 2015). Given that accumulating evidences depict that GFAP can be used for identifying EGCs (Rao et al. 2017), we applied it as a marker for EGCs. Subsequently, the cell tag gene was analyzed in the R language using the “CellMarker” website (http://biocc.hrbmu.edu.cn/CellMarker/), which determines the cell type corresponding to each subpopulation.

### KEGG enrichment analysis

All microarray data were downloaded from the GEO database (http://www.ncbi.nih.gov/geo). The raw data were downloaded as MINiML files. The differentially expressed mRNA was studied using the limma package in the R software. The adjusted *P*-value was analyzed to correct the false positive results in GEO datasets. “Adjusted *P* < 0.05 and Log (Fold Change) > 1 or Log (Fold Change) < − 1” were defined as the threshold for the differential expression of mRNAs. To further confirm the underlying function of potential targets, the data were analyzed by functional enrichment. Kyoto Encyclopedia of Genes and Genomes (KEGG) Enrichment Analysis is a practical resource for studying gene functions and associated high-level genome functional information. To better understand the carcinogenesis of mRNA, ClusterProfiler package (version: 3.18.0) in R was employed to enrich the KEGG pathway.

### Statistical analysis

Statistical analysis was performed using a GraphPad Prism software (version 7.00, USA). The survival rates were analyzed using the log-rank (Mantel-Cox) test and reported as percentages (%). The other data were reported as mean ± standard error of the mean (SEM). Gaussian distribution was evaluated by Shapiro–Wilk normality test. The statistical significance of the differences between groups, except for the survival rate, was evaluated by Student’s t-test or one-way analysis of variance (ANOVA) (Dunnet correction for multiple comparisons). Nonparametric Kruskal–Wallis tests were performed if data did not belong to the Gaussian distribution. Differences were considered significant at a *P* value less than 0.05 (*), less than 0.01 (**), less than 0.001 (***), or less than 0.0001 (****).

## Results

### Inhibition of platelet activation improved intestinal barrier function in septic mice

**Fig. 1:**
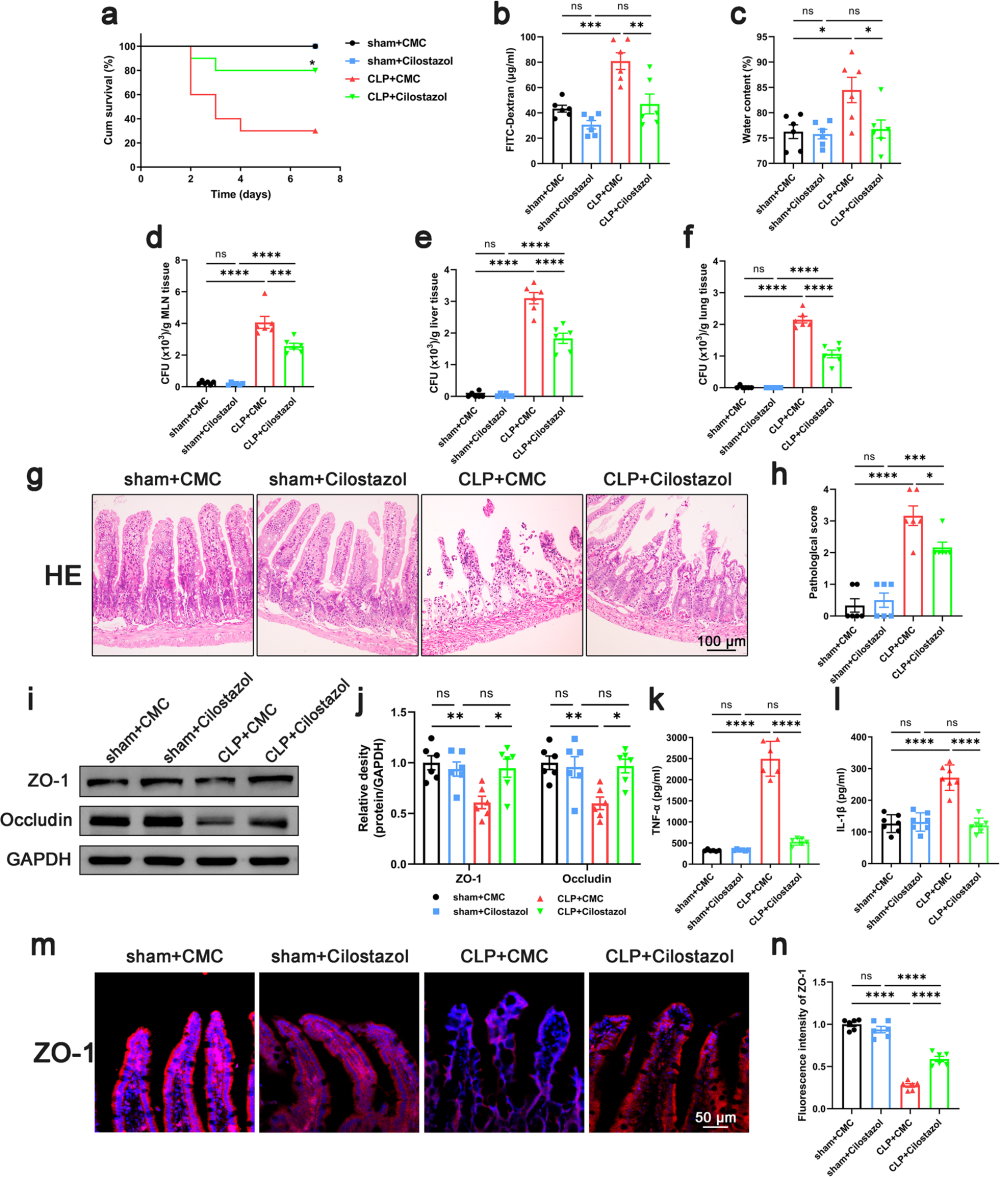
Effect of inhibiting platelet activation on intestinal barrier function in septic mice. Cilostazol (10 mg/kg) was administrated orally 2 h prior to and at 12 h after CLP to inhibit platelet activation. 24 h after CLP, mice were sacrificed, and tissue samples were collected. **a** The survival rate of the mice within 7 days after CLP was observed by survival curves (n = 10). **b**–**f** Intestinal barrier permeability was indicated by serum FITC-Dextran levels (**b**) (n = 6), water content (**c**) of gut (n = 6), and colony-forming units (CFUs) (**d**–**f**) from mesenteric lymph node (MLN), liver and lung (n = 6). **g**, **h** Haematoxylin and eosin (H&E) staining and pathological score, Scale bar = 100 μm (n = 6). **i**, **j** Western blot analysis of ZO-1 and occludin expression (n = 6). **k**, **l** TNF-α and IL-1β levels in intestinal tissues (n = 6). **m**, **n** Immunofluorescence staining analysis of ZO-1 (red), Scale bar = 50 μm (n = 6). The data are presented as the mean ± SEM, *P < 0.05, **P < 0.01, ***P < 0.001, ****P < 0.0001

To confirm the role of platelets in sepsis, KEGG enrichment analysis was performed based on the GSE12624 dataset (35 septic patients vs. 35 healthy person). Data demonstrated an upregulation of Platelet Activation Pathways in the septic patients (Additional file 2: Fig. S2). Based on this evidence, antiplatelet drugs are promising agents to improve the prognosis of sepsis patients (Wang et al. 2018). To study the effect of antiplatelet drug treatment on the intestinal barrier function of septic mice, we applied cilostazol because of its stable effect on platelet inhibition and lower frequency of gastrointestinal bleeding (Barra et al. 2019). The mice were divided into four groups, sham + CMC group, sham + Cilostazol group, CLP + CMC group, and CLP + Cilostazol group. Data demonstrated that the survival rate of mice in CLP + Cilostazol group was significantly increased compared with that in the CLP + CMC group (Fig. 1a). Also, the levels of intestinal permeability and edema decreased significantly in the CLP + Cilostazol group compared to those in the CLP + CMC group (Fig. 1b, c). The results of bacterial load in mesenteric lymph node, lung and liver tissue showed that cilostazol administration significantly reduced the bacterial translocation in septic mice (Fig. 1d–f). Histological analysis of the intestinal showed that the CLP + CMC group had higher histological scores, while the CLP + Cilostazol group showed lower histological scores (Fig. 1g, h). We also observed that the levels of TNF-α and IL-1β in intestinal tissues in the CLP + Cilostazol group were reduced compared with the CLP + CMC group (Fig. 1k, l). But there was no significant difference in the levels of TNF-α and IL-1β in serum between the CLP + CMC group and CLP + Cilostazol group (Additional file 3: Fig. S3). The above results suggested that intestinal barrier function and the local inflammation in septic mice were improved after cilostazol administration. Intestinal barrier function, especially intestinal barrier permeability, was closely related to the content of tight junction proteins in intestinal tissues. Data demonstrated that the expression of tight junction proteins ZO-1 and occludin in intestinal tissues of septic mice was significantly lower than that of sham, while the expression levels of ZO-1 and occludin in the CLP + Cilostazol group were higher than those in the CLP + CMC group (Fig. 1i, j). In the immunofluorescence of intestinal tissue, we also found that the expression of ZO-1 in septic mice was decreased, and the tight junction between epithelial cells became discontinuous, while the expression of ZO-1 in the CLP + Cilostazol group was higher than that in the CLP + CMC group (Fig. 1m, n). To eliminate other potential effects of cilostazol on peripheral blood and cardiac function, CBCs and ultrasound evaluation were performed. Although the count of WBC decreased in septic mice, the administration of cilostazol did not affect the counts of platelet, NEUT, MONO and LYMPH (Additional file 4: Fig. S4). In addition, cilostazol also did not affect cardiac function such as LVEF, LVFS, LVEDD, and LVESD in septic mice (Additional file 5: Fig. S5). Thus, the beneficial effect of cilostazol on intestinal barrier function is not achieved by improving circulation. Taken together, inhibition of platelet activation improved the intestinal barrier function in septic mice.

### Inhibition of platelet activation reduced the release of CD40L and suppressed reactive enteric glia

**Fig. 2:**
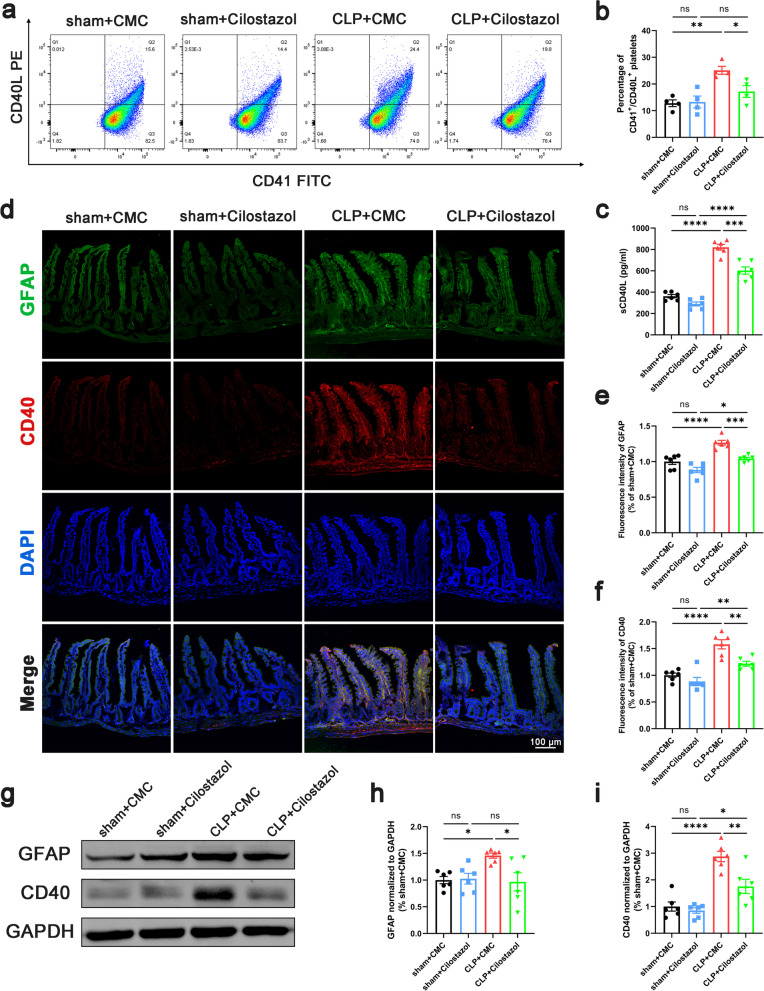
Inhibition of platelet activation suppressed reactive EGCs. **a**, **b** Flow cytometric dot blots of 2-color fluorescence staining of mice platelets with anti-CD40L followed by anti-CD41-FITC. The numbers at corners indicated the percentage of CD41 and CD40L double positive population (n = 4). **c** Serum sCD40L levels (n = 6). **d**–**f** Immunofluorescence staining analysis of GFAP (green) and CD40 (red), Scale bar = 100 μm (n = 6). **g**–**i** Western blot analysis of GFAP and CD40 expression (n = 6). The data are presented as the mean ± SEM, *P < 0.05, **P < 0.01, ***P < 0.001, ****P < 0.0001

**Fig. 3:**
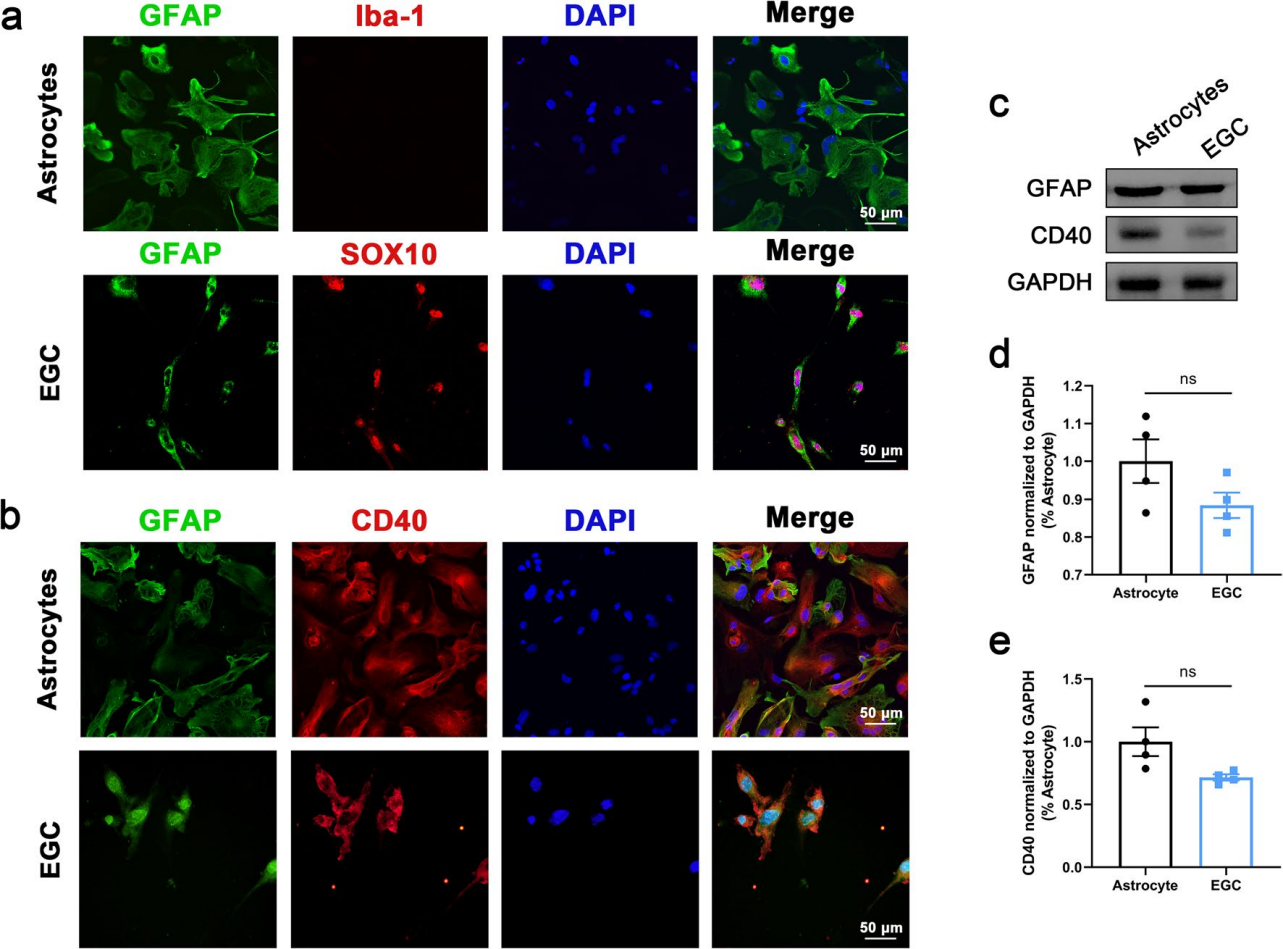
Localization of CD40 in EGCs. **a** The purity of primary EGCs and astrocytes cultures was > 95% as determined by immunofluorescence. **b** GFAP and CD40 immunofluorescence was detected in primary EGCs and astrocytes. **c**–**e** Western blot analysis showed that CD40 was expressed in primary EGCs as in astrocytes

Absent from the surface of resting platelets, CD40L is presented on the platelet surface and released into the circulation rapidly (Lim et al. 2004). Since more than 95% of the circulating CD40L originates from platelet activation (Andre et al. 2002), CD40L has been reported as a biomarker of platelet activation such as PAC-1 and CD62p, and serves as a reliable predictor of sepsis prognosis (Wegrzyn et al. 2021). To investigate the activation of platelets, we detected the concentration of CD40L by ELISA and the proportion of CD40L^+^ platelets by flow cytometry. Data demonstrated that both the concentration of sCD40L and the proportion of CD40L^+^ platelets were increased in the serum of septic mice (Fig. 2a–c). Also, the expression of CD40 (the receptor of CD40L) was upregulated on the intestinal tissue of septic mice (Fig. 2f, g, i). indicating the activation of CD40L-CD40 pathway. EGCs play an important role in the maintenance of the intestinal barrier function (Cheadle et al. 2013; Yu and Li 2014). To identify such group of cell types, a comprehensive analysis of adult mouse distal colon single-cell sequencing data based on GSE156905 dataset gained 17,047 genes and 1384 cells, which had been categorized into 12 individual clusters by UMAP (Additional file 6: Fig. S6a). Considering the approximate expression of GFAP, the marker of EGCs (Rao et al. 2017), between 0 and 5 cluster (Additional file 6: Fig. S6b), the two cluster have been merged into the same cell type as EGCs (Additional file 6: Fig. S6c). Also, our data demonstrated the upregulation of GFAP in intestinal tissue of septic mice (Fig. 2e, g, h). Intriguingly, the expression of GFAP colocalized with CD40 on intestinal tissue, indicating that CD40 is expressed on EGCs (Fig. 2d). To further confirm the localization of CD40 on EGCs, primary EGCs and astrocytes were cultured and purified (the purity of EGCs and astrocytes cultures was always > 95% as determined by immunofluorescence, Fig. 3a). Western blot and immunofluorescence were performed on primary EGCs and their positive control, astrocytes. Data showed that CD40 was localized on EGCs (Fig. 3b–e). In septic mice, after cilostazol application, the expression of GFAP and CD40 decreased accompanied with the decline of CD40L^+^ platelets proportion and CD40L concentration in serum (Fig. 2). Taken together, the protective effect of inhibiting platelet activation may be associated with the suppression of EGC activation at least partially through the CD40L-CD40 signaling pathway.

### CD40L increased inflammatory factor release from EGCs was related to CD40L-CD40-TRAF6 signaling pathway, leading to impaired intestinal barrier integrity

**Fig. 4:**
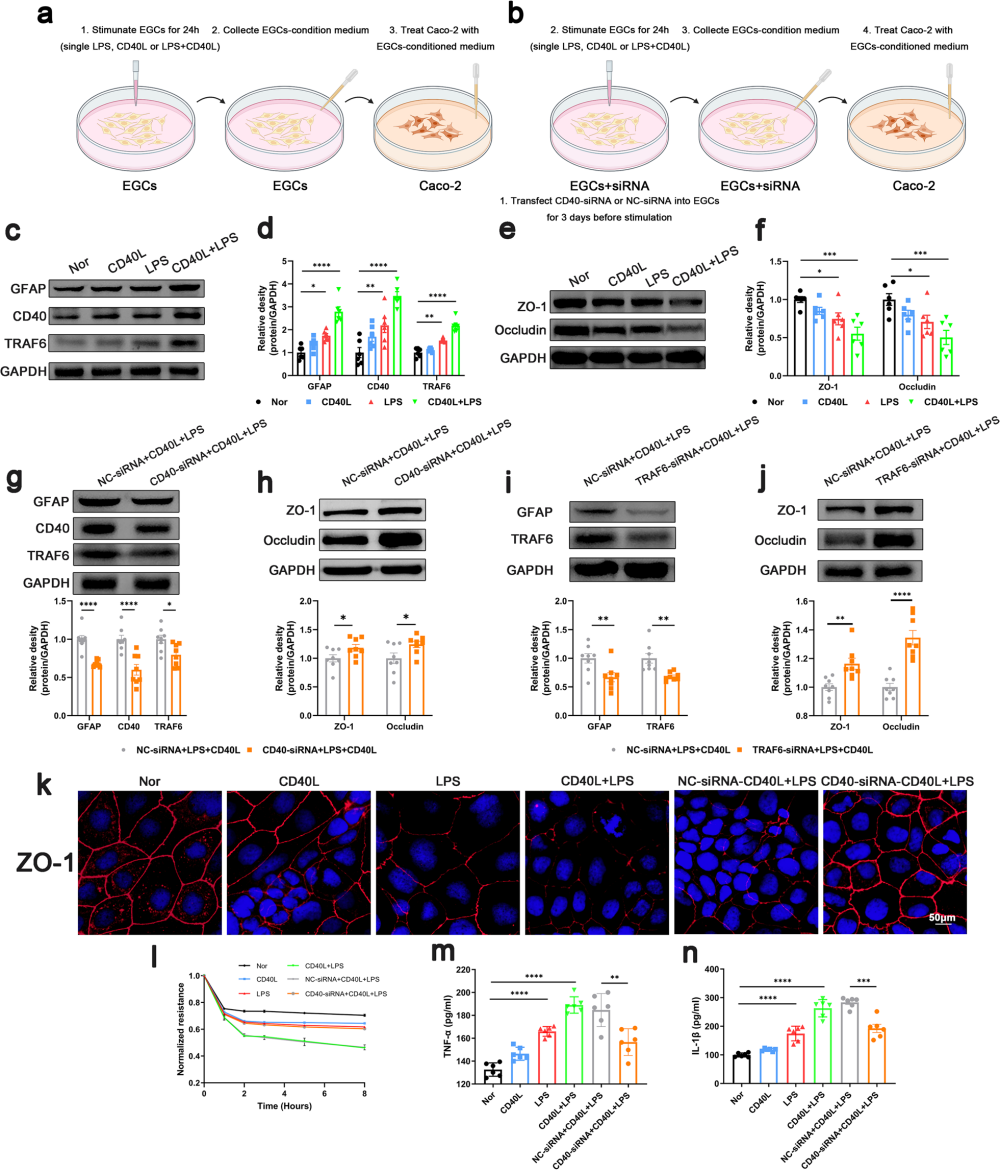
Effect of CD40L-CD40-TRAF6 signaling on EGCs in vitro. **a**, **b** Primary EGCs were stimulated with LPS, CD40L alone or LPS + CD40L for 24 h siRNA transfection and the EGCs-conditioned medium were collected for Caco-2 culture. Western blot analysis of GFAP, CD40 and TRAF6 expression in EGCs (**c**, **d**, **g**, **i**), ZO-1 and occludin expression in Caco-2 cells (**e**, **f**, **h**, **j**) (n = 6). **k** Immunofluorescence staining analysis of ZO-1 (red), Scale bar = 50 μm. **l** Transepithelial electrical resistance (n = 3). TNF-α (**m**) and IL-1β (**n**) levels in EGCs-conditioned medium (n = 6). The data are presented as the mean ± SEM, *P < 0.05, **P < 0.01, ***P < 0.001, ****P < 0.0001

The mechanism of how reactive EGCs disrupt the intestinal barrier function was investigated in vitro. We used LPS and CD40L to stimulate the EGCs and collected the EGCs-conditioned medium for Caco-2 culture (Fig. 4a, b). Data showed that the stimulation of single LPS, CD40L or LPS + CD40L increased the expression of GFAP and CD40 on EGCs. Moreover, the expression of GFAP and CD40 in EGCs stimulated by LPS + CD40L was higher than that stimulated by LPS or CD40L alone (Fig. 4c, d). Given that TRAF family plays a key role in CD40 initiating signaling cascades (Bishop et al. 2007), we examined the changes of TRAF proteins on EGCs stimulated with LPS or LPS + CD40L. Data showed a more pronounced increase in TRAF6 than TRAF 1, 2, 3, 4, 5 (Fig. 4c, Additional file 7: Fig. S7), indicating that TRAF6 is the primary downstream signal of CD40. Next, we cultured the Caco-2 cell with EGCs-conditioned medium to mimic septic epithelium in vivo. We found that conditioned medium from LPS, CD40L alone and LPS + CD40L treated EGCs induced a significant decrease in the expression of ZO-1 and occludin (Fig. 4e, f), reduced the transepithelial electrical resistance (Fig. 4l), and disrupted the integrity of tight junctions (Fig. 4k) in Caco-2 cells. CD40 knockdown in EGCs by CD40-siRNA downregulated the expression of CD40 and GFAP (Fig. 4g). The EGCs-conditioned medium in CD40-siRNA + LPS + CD40L group increased the TEER, the expression of ZO-1 and occludin and the integrity of tight junctions of Caco-2 cells compared with that in the NC-siRNA + LPS + CD40L group (Fig. 4h, k, l). To explore the precise mechanisms by which reactive EGCs disrupted the intestinal barrier function, we measured TNF-α and IL-1β which are closely related to the function of intestinal barrier integrity in the EGCs-conditioned medium. Data showed that the levels of TNF-α and IL-1β elevated under the stimulation of LPS, CD40L alone or LPS + CD40L, while CD40 knockdown in EGCs by CD40-siRNA decreased the levels of TNF-α and IL-1β compared to the NC-siRNA + LPS + CD40L group (Fig. 4m, n). Subsequently, we used TRAF6-siRNA to downregulate TRAF6 and observed the same results in CD40-siRNA treatment (Fig. 4i, j). Taken together, CD40L-CD40 signaling pathway participates in the activation of EGCs during sepsis and affects the release of inflammatory factors TNF-α and IL-1β of EGCs, thereby affecting the intestinal barrier permeability.

### Blocking CD40L-CD40-TRAF6 signaling pathway improved intestinal barrier function in septic mice

**Fig. 5:**
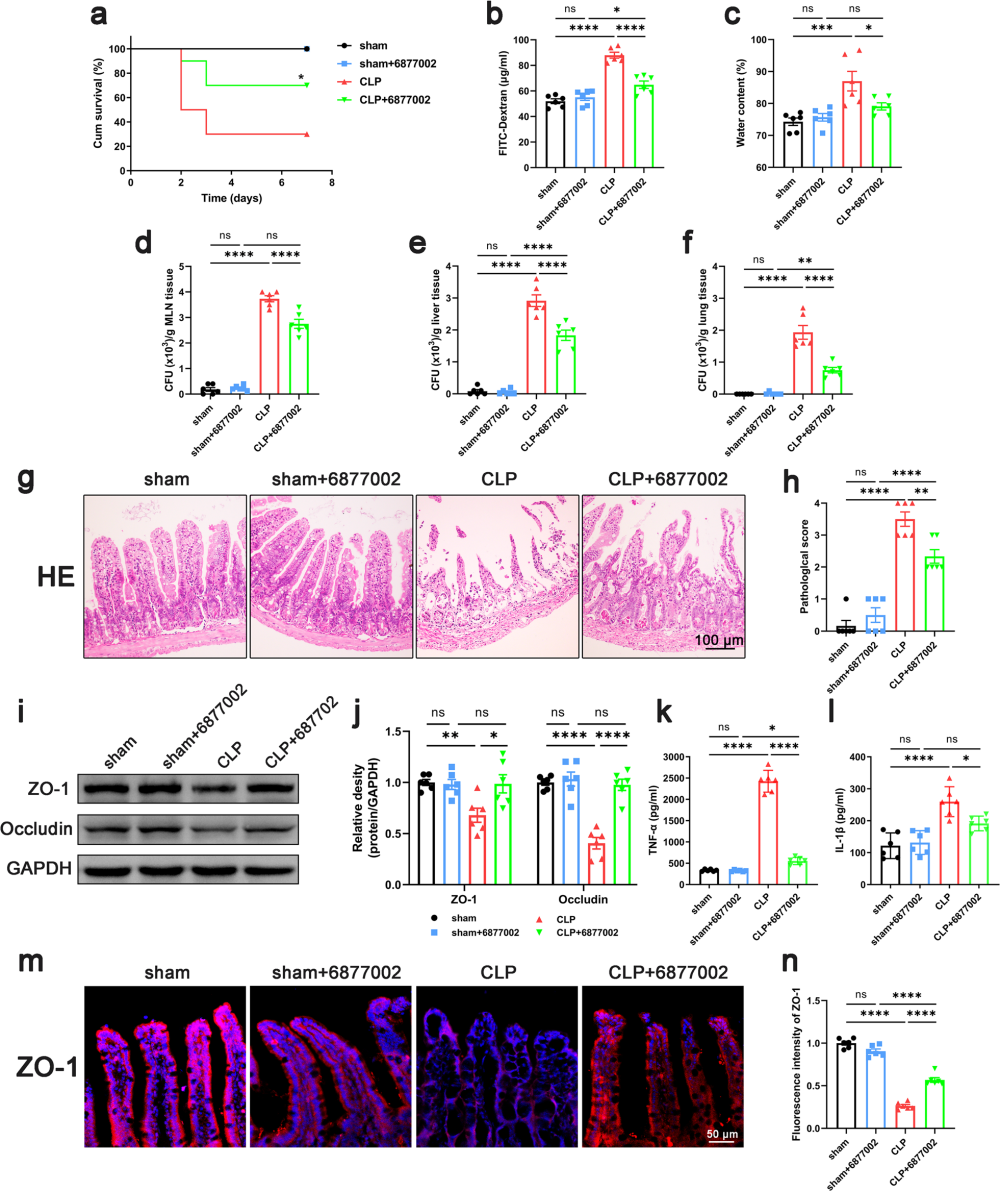
Blocking CD40L-CD40-TRAF6 signaling pathway improved intestinal barrier function in septic mice. Compound 6877002 (10 μmol/kg) was injected intraperitoneally 2 h prior to and at 12 h after CLP to block the CD40-TRAF6 signaling pathway. 24 h after CLP, mice were sacrificed, and tissue samples were collected. **a** The survival percentage of the mice was investigated within 7 days after CLP by survival curves (n = 10). Intestinal barrier permeability was indicated by serum FITC-Dextran levels (**b**) (n = 6), water content (**c**) of gut (n = 6), and colony-forming units (CFUs) (**d**–**f**) from mesenteric lymph node (MLN), liver and lung (n = 6). **g**, **h** Haematoxylin and eosin (H&E) staining and pathological score, Scale bar = 100 μm (n = 6). **i**, **j** Western blot analysis of ZO-1 and occludin expression (n = 6). **k**, **l** TNF-α and IL-1β levels in intestinal tissues (n = 6). **m**, **n** Immunofluorescence staining analysis of ZO-1 (red), Scale bar = 50 μm (n = 6). The data are presented as the mean ± SEM, *P < 0.05, **P < 0.01, ***P < 0.001, ****P < 0.0001

To verify the role of CD40L-CD40-TRAF6 signaling pathway, we used the compound 6877002, a CD40-TRAF6 inhibitor, to block CD40-TRAF6 signaling pathway in vivo. Data showed that compound 6877002 improved the survival rate of CLP mice (Fig. 5a), and reduced intestinal permeability, the degree of intestinal edema and bacterial translocation (Fig. 5b–f). Histological analysis of the intestinal showed that the 6877002-treated septic mice had lower histological scores compared with the CLP group (Fig. 5g, h). And the levels of TNF-α and IL-1β in intestinal tissues were reduced in 6877002-treated septic mice compared with the CLP group (Fig. 5k, l). Moreover, after compound 6877002 treatment, the expression level of ZO-1 and occludin was significantly higher than that of CLP (Fig. 5i, j), and immunofluorescence staining also showed that the continuity was restored and expression of ZO-1 protein were improved (Fig. 5m, n). These results suggested that blocking CD40L-CD40-TRAF6 signaling pathway may be involved in the efficacy of cilostazol in ameliorating intestinal barrier dysfunction.

### GSNO supplementation mitigated the intestinal barrier dysfunction in septic mice

**Fig. 6:**
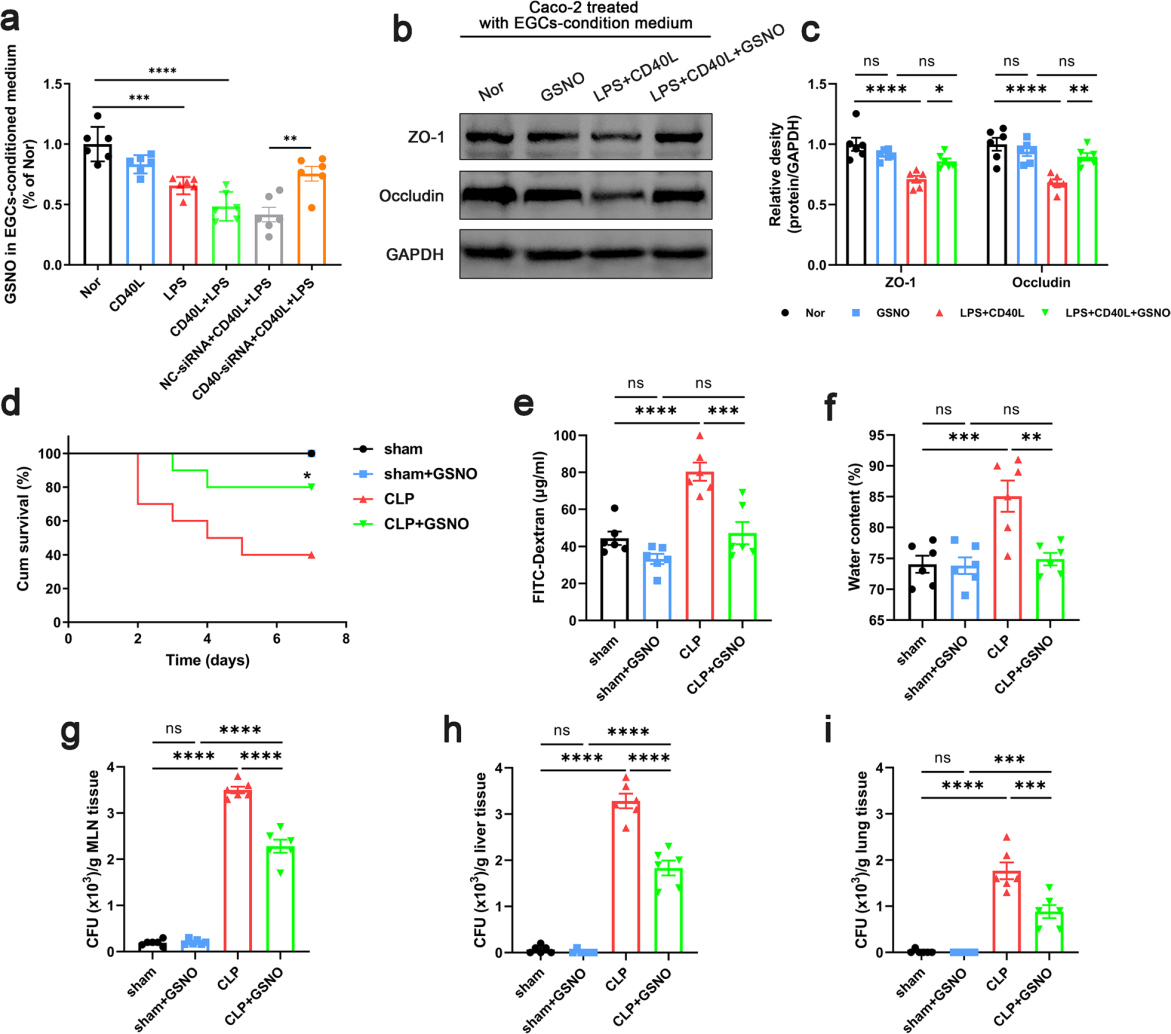
Effect of GSNO pretreatment on intestinal epithelial barrier. In vitro, EGCs-conditioned medium was collected to measure the GSNO level (**a**) (n = 6). The levels of ZO-1 and occludin expression in Caco-2 cells were evaluated by western blot after the stimulation of EGCs-conditioned medium with or without GSNO 50 µM) supplementation (**b**, **c**) (n = 6). In vivo, GSNO was administrated intraperitoneally at 10 mg/kg/day for 7 days before CLP. 24 h after CLP, mice were sacrificed, and tissue samples were collected. **d** The survival percentage of the mice was investigated within 7 days after CLP by survival curves (n = 10). Intestinal barrier permeability was indicated by serum FITC-Dextran levels (**e**) (n = 6), water content (**f**) of gut (n = 6), and colony-forming units (CFUs) (**g**–**i**) from mesenteric lymph node (MLN), liver and lung (n = 6). The data are presented as the mean ± SEM, *P < 0.05, **P < 0.01, ***P < 0.001, ****P < 0.0001

EGCs were reported to maintain intestinal epithelial tight junction by secreting GSNO directly (Neunlist et al. 2014; Savidge et al. 2007). We observed that the EGCs released less GSNO under the stimulation of LPS, CD40L alone or LPS + CD40L (Fig. 6a), which shows the same tendency as the integrity of the epithelial barrier (Fig. 4k, l). Likewise, the administration of GSNO increased the expression of ZO-1 and occludin on the epithelial barrier (Fig. 6b, c). To study the effect of GSNO on the intestinal barrier dysfunction of septic mice, it was administrated before CLP. With the supplementation of GSNO, the survival rate of septic mice was increased, while the intestinal permeability, the degree of intestinal edema and bacterial translocation were alleviated (Fig. 6d–i). These results suggested that GSNO administration mitigated the intestinal barrier dysfunction of septic mice.

## Discussion

**Fig. 7:**
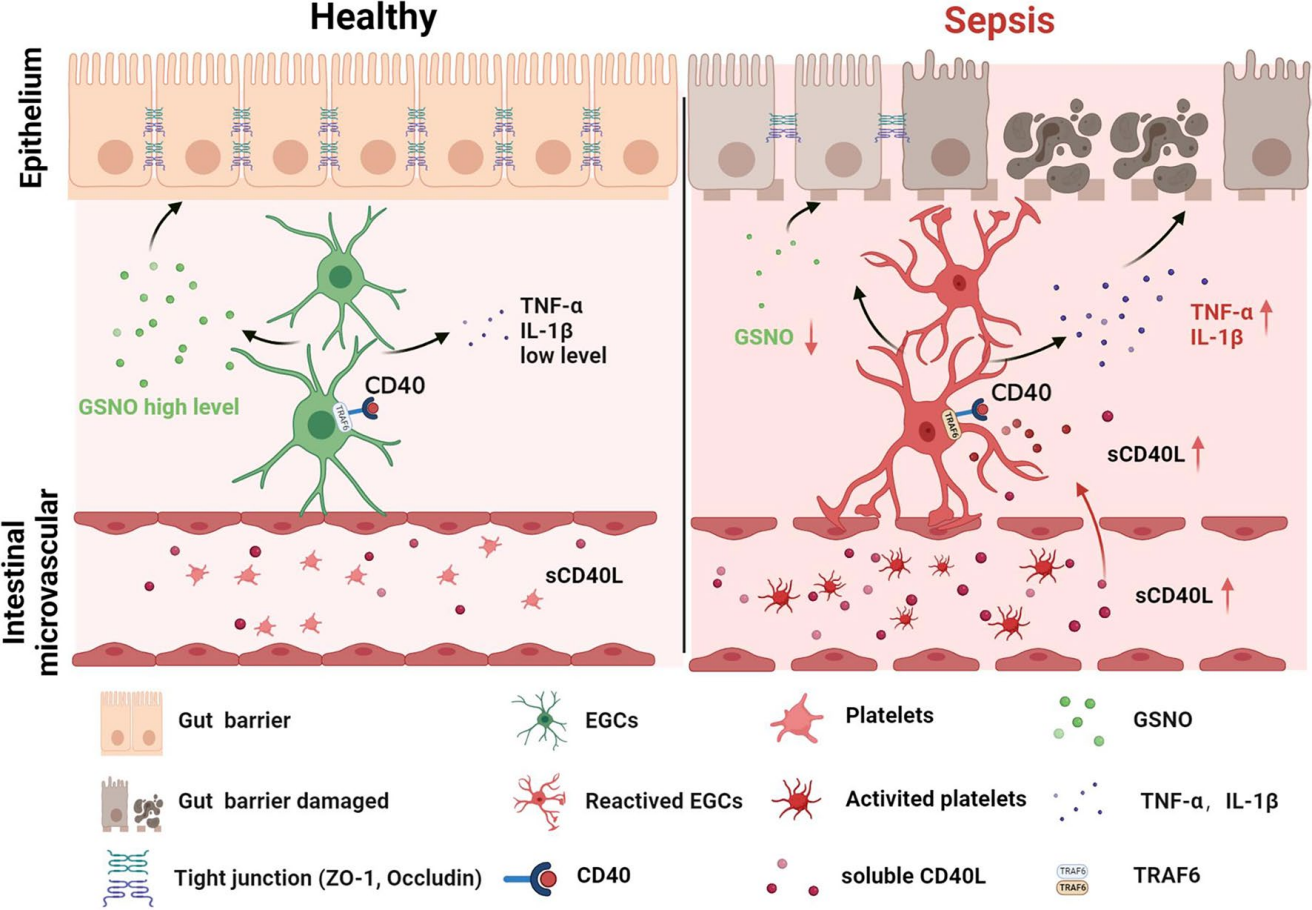
Graphic summary illustration. Inhibition of platelet activation suppress reactive enteric glia and mitigated intestinal barrier dysfunction during sepsis, which is associated with CD40L-CD40 signaling pathway

Platelets are activated and trapped in the intestine in the early stage of sepsis, which is associated with intestinal barrier dysfunction and increased mortality rate in sepsis (Sigurdsson et al. 1992). The present study demonstrated that EGCs expressed CD40 and could be activated by platelet-derived CD40L in septic mice. Due to increased TNF-α, IL-1β and decreased GNSO secreted from reactive EGCs, intestinal epithelial tight junction disrupted and induced intestinal barrier dysfunction. On the contrary, inhibition of platelet activation reduced the plasma sCD40L level and suppressed EGCs activation. Consequently, the restoration of EGC function resulted in sufficient GSNO and reduced TNF-α and IL-1β, which were related to effectuating the repair of tight junction disruption, reducing the intestinal barrier permeability, and increasing the survival rate of septic mice. Such results were similarly observed in septic mice treated with compound 6877002, a CD40-TRAF6 inhibitor, indicating that CD40L-CD40-TRAF6 signaling pathway plays an important role in platelets activating EGCs and disturbing the intestinal barrier function (Fig. 7). Therefore, our research provided further evidence that inhibition of platelet activation in the early stage of sepsis may be a prospective strategy for sepsis treatment.

Despite its coagulation function, increasing evidence showed that platelets were involved in the regulation of inflammation and barrier function during sepsis. Clinical evidence suggested that inhibition of platelet activation may improve the prognosis of patients with sepsis (Sigurdsson et al. 1992; Wang et al. 2018). Blockade of platelet GPIb receptor by anfibatide attenuated blood–brain barrier disruption through reduced microthrombosis (Chen et al. 2018). Similarly, activated platelets were the key effector in increasing alveolar capillary permeability and inducing diffuse alveolar damage in acute respiratory distress syndrome (Middleton et al. 2016). Although widely used in the clinic among the numerous platelet activation inhibitors, clopidogrel and aspirin usually have significant gastrointestinal side effects and bleeding risk (Eikelboom et al. 2012). Therefore, we utilized cilostazol, because of its low risk of gastrointestinal bleeding, stable effect on platelet inhibition (Barra et al. 2019), and increased survival in endotoxemic mice (Chang 2015). After cilostazol application, we observed that the expression of ZO-1 and occludin was increased significantly, the integrity of tight junctions was restored, and intestinal barrier permeability was decreased. In line with the previous study (Chang 2015), our data also demonstrated that inhibition of platelet activation improves the prognosis and survival rate of septic mice. Additionally, we observed that the efficacy of cilostazol was based on the amelioration of intestinal barrier dysfunction.

In addition to inhibiting platelet activation, cilostazol also enhances vasodilatation and cardiac contractility (Sun et al. 2007), prevents intimal hyperplasia and proliferation of vascular smooth muscle cells, lowers blood pressure, decreases inflammation (Park et al. 2010) and increases cerebral blood flow, improves myelin repair, and strengthens astrocyte-to-neuron energy supply (de Havenon et al. 2021). Therefore, the question of concern is whether the beneficial effect of cilostazol comes from the inhibition of platelet activation rather than other effects? Experimental and literature evidences were used to eliminate the indirect effect described above. First, with the help of ultrasonography, we did not find significant cardiac function change in septic mice with or without cilostazol treatment. Second, cilostazol may enhance vasodilatation and lower blood pressure. Indeed, septic shock is characterized by hyperdynamic circulation, i.e., low systemic vascular resistance and high cardiac output (Uriu et al. 2002). The enhanced vasodilatation and lower blood pressure may exacerbate shock and decrease survival rate. Given that the compensatory mechanisms of shock is to redistribute blood flow to preserve heart and brain circulation by expensing skin, kidney and splanchnic circulation (Perner and Backer 2014), the intestinal barrier is more likely to be impaired than to benefit from these effect of cilostazol. Third, cilostazol decreased the level of inflammation factors in intestine but did not affect their level in the serum, suggesting that this beneficial effect might come from the intestine rather than the alleviation of systemic inflammatory response. Thus, the beneficial effect is more likely to come mainly from inhibition of platelet activation.

Recent studies have shown that CD40 and CD40L are significantly elevated in sepsis patients and model animals, which are closely related to the severity of sepsis (Gold et al. 2003; Liang et al. 2021; Lorente et al. 2017, 2011). The CD40/CD40L interaction has been regarded as an important participant in immune and inflammation responses (Tang et al. 2021). Typically, the intestine is complex in component, and most immune cells expressing CD40, such as T cells, B cells, basophils, eosinophils, monocytes/macrophages and dendritic cells (Liu et al. 1999), can respond to CD40L. Septic patients showed increased monocyte expression of CD40 compared with healthy control subjects. Also, anti-CD40 treatment increased the Bcl-x level in splenic B and T cells as well as in thymic T cells, thereby attenuating sepsis (Liu et al. 2018). Besides immune cells, emerging evidence has demonstrated that CD40 is also expressed on glial cells, such as astrocytes and microglia, and CD40L mediates the activation of astrocytes and microglia through platelet CD40L, contributing to the neuroinflammation and leaky blood–brain barrier (Bhat et al. 2017; Michels et al. 2015). Therefore, we hypothesize that CD40 may be expressed on EGCs and related to the intestinal barrier dysfunction during sepsis, because EGCs share many characteristics with astrocytes and are associated with intestinal epithelial cells (Jessen and Mirsky 2005). According to our findings, CD40 is localized on the membrane of EGCs and overexpressed during sepsis both in CLP model and LPS-induced cell model, following the increased expression of GFAP. We also observed that CD40L^+^ platelets were increased in septic mice, while cilostazol inhibited the platelet activation and reduced the proportion of CD40L^+^ platelets in serum. These results indicated that cilostazol mitigated intestinal barrier dysfunction by inhibiting the platelet activation and was associated with the CD40L-CD40 signaling pathway of enteric glial cells.

To confirm whether CD40L-CD40 is a key signaling pathway regulate EGC activation during sepsis and find the primary downstream signal of CD40L-CD40, specific inhibitors are required to block CD40L-CD40 signaling pathway. Monoclonal antibody against CD40L may be the ideal inhibitor to block the CD40L-CD40 signaling pathway completely, but evidence shows that it leads to severe thromboembolic complications (Kawai et al. 2000). The canonical CD40L-CD40 signaling pathway is mediated by the recruitment and activation of TRAFs including TRAF1, 2, 3, 4, 5 and TRAF6. TRAF6 is a central regulator of sepsis (Lalani et al. 2018), and blocking CD40-TRAF6 pathway while leaving CD40-TRAF 2, 3, 5 pathways functional would cause less severe immune-suppressive side effects. Some studies showed that common genetic variants in TRAF6 were significantly associated with susceptibility to sepsis-induced acute lung injury (Song et al. 2012) and peripheral blood level of TRAF6 in SAE patients was elevated and related to the severity of SAE (Zhang et al. 2016). Our in vitro experiment demonstrated that TRAF6 changed more significantly than other TRAF proteins under the treatment of LPS and CD40L, and silencing TRAF6 could inhibit EGCs activation and ameliorate Caco-2 cell damage induced by conditioned medium of LPS + CD40L-stimulated EGCs. Accordingly, we administrated the compound 6877002, an effective CD40-TRAF6 signaling inhibitor, to investigate whether blocking the CD40L-CD40-TRAF6 signaling would save the intestinal barrier dysfunction from reactive EGCs. Our data showed that the effect of blocking CD40L-CD40-TRAF6 signaling pathway was comparable with that of cilostazol by restoring the disrupted continuous tight junctions between intestinal epithelial cells, reducing the intestinal barrier permeability, decreasing the levels of inflammatory factors, and increasing the survival rate of septic mice. Thus, CD40L-CD40-TRAF6 signaling pathway may be involved in signal between platelets and EGCs in regulating intestinal barrier function.

EGCs are closely associated with intestinal epithelial cells and play a major role in maintaining the intestinal barrier function (Savidge et al. 2007; Vergnolle and Cirillo 2018). The ablation of EGCs in transgenic mice causes intestinal barrier dysfunction, associated with the decreased release of EGCs-derived GSNO (Savidge et al. 2007). Herein, we observed that Caco-2 monolayer integrity and the expression levels of ZO-1 and occludin are related to the levels of GSNO and inflammation factors, which is consistent with previous studies (Cheadle et al. 2013; Savidge et al. 2007). In addition, our results showed that GSNO supplementation restored intestinal function and increased the survival rates of septic mice, which in turn reduced the levels of TNF-α and IL-1β in plasma and intestinal tissues (Li et al. 2016). Above all, our research provided further evidence that inhibition of platelet activation suppressed reactive enteric glia related to CD40L-CD40-TRAF6 signaling pathway and affected the release of GSNO and inflammatory factors TNF-α and IL-1β of enteric glial cells, thereby mitigating intestinal barrier dysfunction during sepsis.

However, we are aware that our study may have two limitations. First, we didn’t perform the experiments on CD40 KO mice and TRAF6 KO mice, which can provide stronger direct evidence of causation between cilostazol and CD40L-CD40-TRAF6 signaling pathway. Another limitation is that the survival benefits of cilostazol may result from other effects, we cannot deny the other indirect beneficial effect of cilostazol in vivo. Despite these limitations, our findings identify the relationship between the effect of cilostazol on platelet inhibition and amelioration of intestinal barrier dysfunction, which indicated that inhibition of platelet activation maybe a potential therapeutic strategy and a promising target for sepsis.

## Conclusions

In summary, we identify the correlation between inhibition of platelet activation and amelioration of intestinal barrier dysfunction during sepsis, which is associated with the suppression of CD40L-CD40-TRAF6 signaling pathway and the reduction of EGC activation. These findings indicated that inhibition of platelet activation maybe a potential therapeutic strategy and a promising target for sepsis.

## Supplementary Information

**Additional file 1: Figure S1.** The gating strategies for the flow cytometry experiments. Data analysis was performed using FlowJo (Ashland, OR). Scatter and staining with the FITC-anti-CD41 and PE-anti-CD40L antibodies were used to gate platelet population. Cells were first gated by regions within a side scatter area (SSC-A) versus forward scatter area (FSC-A) plot, and then through gating those populations in the SSC-A versus FITC-A plots. Activated platelets were defined as FITC-anti-CD41-A positive and PE-anti-CD40L-A positive.

**Additional file 2: Figure S2.** KEGG enrichment of differential expressed genes in septic patients compared with healthy person. **a** The volcano plot was constructed using the fold change values and P-adjust. Red dots indicate upregulated genes; blue dots indicate downregulated genes. **b** The heatmap of the differential gene expression, where different colors represent trends of gene expression in different tissues. **c**, **d** The enriched KEGG signaling pathways were selected to demonstrate the primary biological actions of major potential mRNA. Colors represent the significance of differential enrichment; the size of the circles represents the number of genes. In the enrichment result, *P* < 0.05 or FDR < 0.05 is considered to be a meaningful pathway (enrichment score with − log10 (*P*) of more than 1.3).

**Additional file 3: Figure S3.** Effects of cilostazol treatment on serum inflammatory factors. The level of serum TNF-α (**a**) and IL-1β (**b**) in each group (n = 6). The data are presented as the mean ± SEM, ***P < 0.001, ****P < 0.0001, and ns indicates no significant difference.

**Additional file 4: Figure S4.** Effects of cilostazol treatment on complete blood counts after CLP. **a, b** The level of platelets (**a**), WBC (**b**), NEUT (**c**), MONO (**d**) and LYMPH (**e**) (n = 5). WBC, white blood cells; NEUT, neutrophil; MONO, monocyte; LYMPH, lymphocyte. The data are presented as the mean ± SEM, *P < 0.05, ***P < 0.001, and ns indicates no significant difference.

**Additional file 5: Figure S5.** Effect of cilostazol treatment on cardiac function after CLP. **a** Representative M-mode images of the four different groups. **b**–**e** Quantitative analysis of LVEF (**b**), LVFS (**c**), LVEDD (**d**), and LVESD (**e**) in each group (n = 6). These data indicate no significant difference.

**Additional file 6: Figure S6.** Single-cell sequencing analysis of adult mice intestine. **a** Dotplot for highest specificity gene markers of cell clusters. Dot size represents the percentage of cells expressing the denoted gene, and the color represents average normalized expression level within the denoted cluster. **b** The expression of GFAP in different cell clusters by analysis of the Uniform Manifold Approximation and Projection (UMAP). **c** Different cell subpopulation of the GSE156905 dataset derived from the adult mice intestine.

**Additional file 7: Figure S7.** Effect of LPS and CD40L on TRAFs proteins in EGCs. **a** Representative western blot images of TRAFs proteins expression in EGCs. **b**–**g** Western blot analysis of TRAF1 (**b**), TRAF2 (**c**), TRAF3 (**d**), TRAF4 (**e**), TRAF5 (**f**) and TRAF6 (**g**) expression in different groups of EGCs (n = 6). The data are presented as the mean ± SEM, *P < 0.05, **P < 0.01, ***P < 0.001, ****P < 0.0001.

## Data Availability

The datasets used and/or analyzed during the current study are available from the corresponding author on reasonable request.

## Abbreviations

MODS: Multiple organ dysfunction syndrome
EGCs: Enteric glial cells
LPS: Lipopolysaccharide
CLP: Cecal ligation and puncture
sCD40L: Soluble CD40 ligand
GSNO: *S*-Nitrosoglutathione
CFU: Colony forming units
CMC: Carboxymethyl cellulose sodium salt
LVEDD: Left ventricular end diastolic diameter
LVESD: Left ventricular end systolic dimension
LVEF: Left ventricular ejection fraction
LVFS: Left ventricular fractional shortening
WBC: White blood cells
NEUT: Neutrophil
MONO: Monocyte
LYMPH: Lymphocyte

## References

[CR1] Andre P, Nannizzi-Alaimo L, Prasad SK, Phillips DR (2002). Platelet-derived CD40L: the switch-hitting player of cardiovascular disease. Circulation.

[CR2] Armacki M, Trugenberger AK, Ellwanger AK, Eiseler T, Schwerdt C, Bettac L (2018). Thirty-eight-negative kinase 1 mediates trauma-induced intestinal injury and multi-organ failure. J Clin Invest.

[CR3] Barra ME, Berger K, Tesoro EP, Brophy GM (2019). Periprocedural neuroendovascular antiplatelet strategies for thrombosis prevention in clopidogrel-hyporesponsive patients. Pharmacotherapy.

[CR4] Baumgart DC, Dignass AU (2002). Intestinal barrier function. Curr Opin Clin Nutr Metab Care.

[CR5] Bhat SA, Goel R, Shukla R, Hanif K (2017). Platelet CD40L induces activation of astrocytes and microglia in hypertension. Brain Behav Immun.

[CR6] Bi J, Zhang J, Ren Y, Du Z, Li T, Wang T (2020). Irisin reverses intestinal epithelial barrier dysfunction during intestinal injury via binding to the integrin alphaVbeta5 receptor. J Cell Mol Med.

[CR7] Bishop GA, Moore CR, Xie P, Stunz LL, Kraus ZJ (2007). TRAF proteins in CD40 signaling. Adv Exp Med Biol.

[CR8] Blikslager AT, Moeser AJ, Gookin JL, Jones SL, Odle J (2007). Restoration of barrier function in injured intestinal mucosa. Physiol Rev.

[CR9] Chang KC (2015). Cilostazol inhibits HMGB1 release in LPS-activated RAW 264.7 cells and increases the survival of septic mice. Thromb Res.

[CR10] Cheadle GA, Costantini TW, Lopez N, Bansal V, Eliceiri BP, Coimbra R (2013). Enteric glia cells attenuate cytomix-induced intestinal epithelial barrier breakdown. PLoS ONE.

[CR11] Chelakkot C, Ghim J, Ryu SH (2018). Mechanisms regulating intestinal barrier integrity and its pathological implications. Exp Mol Med.

[CR12] Chen C, Li T, Zhao Y, Qian Y, Li X, Dai X (2018). Platelet glycoprotein receptor Ib blockade ameliorates experimental cerebral ischemia-reperfusion injury by strengthening the blood-brain barrier function and anti-thrombo-inflammatory property. Brain Behav Immun.

[CR13] de Havenon A, Sheth KN, Madsen TE, Johnston KC, Turan TN, Toyoda K (2021). Cilostazol for secondary stroke prevention: history, evidence, limitations, and possibilities. Stroke.

[CR14] Dewitte A, Lepreux S, Villeneuve J, Rigothier C, Combe C, Ouattara A (2017). Blood platelets and sepsis pathophysiology: a new therapeutic prospect in critically [corrected] ill patients?. Ann Intensive Care.

[CR15] Dominguez JA, Samocha AJ, Liang Z, Burd EM, Farris AB, Coopersmith CM (2013). Inhibition of IKKbeta in enterocytes exacerbates sepsis-induced intestinal injury and worsens mortality. Crit Care Med.

[CR16] Eikelboom JW, Hirsh J, Spencer FA, Baglin TP, Weitz JI (2012). Antiplatelet drugs: Antithrombotic therapy and prevention of thrombosis, 9th ed: American college of chest physicians evidence-based clinical practice guidelines. Chest.

[CR17] Gold JA, Parsey M, Hoshino Y, Hoshino S, Nolan A, Yee H (2003). CD40 contributes to lethality in acute sepsis: in vivo role for CD40 in innate immunity. Infect Immun.

[CR18] Grubisic V, Gulbransen BD (2017). Enteric glia: the most alimentary of all glia. J Physiol.

[CR19] Gulbransen B, Sharkey KA (2012). Novel functional roles for enteric glia in the gastrointestinal tract. Nat Rev Gastroenterol Hepatol.

[CR20] Hu Q, Ren H, Li G, Wang D, Zhou Q, Wu J (2019). STING-mediated intestinal barrier dysfunction contributes to lethal sepsis. EBioMedicine.

[CR21] Jessen K, Mirsky R (1980). Glial cells in the enteric nervous system contain glial fibrillary acidic protein. Nature.

[CR22] Jessen KR, Mirsky R (2005). The origin and development of glial cells in peripheral nerves. Nat Rev Neurosci.

[CR23] Kawai T, Andrews D, Colvin RB, Sachs DH, Cosimi AB (2000). Thromboembolic complications after treatment with monoclonal antibody against CD40 ligand. Nat Med.

[CR24] Klaus DA, Motal MC, Burger-Klepp U, Marschalek C, Schmidt EM, Lebherz-Eichinger D (2013). Increased plasma zonulin in patients with sepsis. Biochem Med.

[CR25] Lalani AI, Zhu S, Gokhale S, Jin J, Xie P (2018). TRAF molecules in inflammation and inflammatory diseases. Curr Pharmacol Rep.

[CR26] Li Z, Zhang X, Zhou H, Liu W, Li J (2016). Exogenous S-nitrosoglutathione attenuates inflammatory response and intestinal epithelial barrier injury in endotoxemic rats. J Trauma Acute Care Surg.

[CR27] Li Y, Wang Y, Chang H, Cheng B, Miao J, Li S (2021). Inhibitory effects of dexmedetomidine and propofol on gastrointestinal tract motility involving impaired enteric glia Ca(2+) response in mice. Neurochem Res.

[CR28] Liang Y, Zhu C, Sun Y, Li Z, Wang L, Liu Y (2021). Persistently higher serum sCD40L levels are associated with outcome in septic patients. BMC Anesthesiol.

[CR29] Lim HS, Blann AD, Lip GY (2004). Soluble CD40 ligand, soluble P-selectin, interleukin-6, and tissue factor in diabetes mellitus: relationships to cardiovascular disease and risk factor intervention. Circulation.

[CR30] Liu Z, Colpaert S, D'Haens GR, Kasran A, de Boer M, Rutgeerts P (1999). Hyperexpression of CD40 ligand (CD154) in inflammatory bowel disease and its contribution to pathogenic cytokine production. J Immunol.

[CR31] Liu ZL, Hu J, Xiao XF, Peng Y, Zhao SP, Xiao XZ (2018). The CD40 rs1883832 polymorphism affects sepsis susceptibility and sCD40L levels. Biomed Res Int.

[CR32] Lorente L, Martin MM, Varo N, Borreguero-Leon JM, Sole-Violan J, Blanquer J (2011). Association between serum soluble CD40 ligand levels and mortality in patients with severe sepsis. Crit Care.

[CR33] Lorente L, Martin MM, Perez-Cejas A, Ferreres J, Sole-Violan J, Labarta L (2017). Non-survivor septic patients have persistently higher serum sCD40L levels than survivors. J Crit Care.

[CR34] Ma C, Fu Q, Diggs LP, McVey JC, McCallen J, Wabitsch S (2022). Platelets control liver tumor growth through P2Y12-dependent CD40L release in NAFLD. Cancer Cell.

[CR35] Michels M, Danieslki LG, Vieira A, Florentino D, Dall'Igna D, Galant L (2015). CD40-CD40 ligand pathway is a major component of acute neuroinflammation and contributes to long-term cognitive dysfunction after sepsis. Mol Med.

[CR36] Middleton EA, Weyrich AS, Zimmerman GA (2016). Platelets in pulmonary immune responses and inflammatory lung diseases. Physiol Rev.

[CR37] Neunlist M, Rolli-Derkinderen M, Latorre R, Van Landeghem L, Coron E, Derkinderen P (2014). Enteric glial cells: recent developments and future directions. Gastroenterology.

[CR38] Obermuller B, Frisina N, Meischel M, Singer G, Stanzl-Tschegg S, Lichtenegger H (2020). Examination of intestinal ultrastructure, bowel wall apoptosis and tight junctions in the early phase of sepsis. Sci Rep.

[CR39] Otani S, Oami T, Yoseph BP, Klingensmith NJ, Chen CW, Liang Z (2020). Overexpression of BCL-2 in the intestinal epithelium prevents sepsis-induced gut barrier dysfunction via altering tight junction protein expression. Shock.

[CR40] Park WS, Jung WK, Lee DY, Moon C, Yea SS, Park SG (2010). Cilostazol protects mice against endotoxin shock and attenuates LPS-induced cytokine expression in RAW 264.7 macrophages via MAPK inhibition and NF-kappaB inactivation: not involved in cAMP mechanisms. Int Immunopharmacol.

[CR41] Perner A, De Backer D (2014). Understanding hypovolaemia. Intensive Care Med.

[CR42] Peterson LW, Artis D (2014). Intestinal epithelial cells: regulators of barrier function and immune homeostasis. Nat Rev Immunol.

[CR43] Rao M, Rastelli D, Dong L, Chiu S, Setlik W, Gershon M (2017). Enteric glia regulate gastrointestinal motility but are not required for maintenance of the epithelium in mice. Gastroenterology.

[CR44] Rittirsch D, Huber-Lang MS, Flierl MA, Ward PA (2009). Immunodesign of experimental sepsis by cecal ligation and puncture. Nat Protoc.

[CR45] Rosenbaum C, Schick MA, Wollborn J, Heider A, Scholz CJ, Cecil A (2016). Activation of myenteric glia during acute inflammation in vitro and in vivo. PLoS ONE.

[CR46] Savidge TC, Newman P, Pothoulakis C, Ruhl A, Neunlist M, Bourreille A (2007). Enteric glia regulate intestinal barrier function and inflammation via release of *S*-nitrosoglutathione. Gastroenterology.

[CR47] Schneider S, Wright CM, Heuckeroth RO (2019). Unexpected roles for the second brain: enteric nervous system as master regulator of bowel function. Annu Rev Physiol.

[CR48] Sigurdsson GH, Christenson JT, El-Rakshy MB, Sadek S (1992). Intestinal platelet trapping after traumatic and septic shock. An early sign of sepsis and multiorgan failure in critically ill patients?. Crit Care Med.

[CR49] Song Z, Yao C, Yin J, Tong C, Zhu D, Sun Z (2012). Genetic variation in the TNF receptor-associated factor 6 gene is associated with susceptibility to sepsis-induced acute lung injury. J Transl Med.

[CR50] Sun B, Li H, Shakur Y, Hensley J, Hockman S, Kambayashi J (2007). Role of phosphodiesterase type 3A and 3B in regulating platelet and cardiac function using subtype-selective knockout mice. Cell Signal.

[CR51] Tang T, Cheng X, Truong B, Sun L, Yang X, Wang H (2021). Molecular basis and therapeutic implications of CD40/CD40L immune checkpoint. Pharmacol Ther.

[CR52] Uriu K, Osajima A, Hiroshige K, Watanabe H, Aibara K, Inada Y (2002). Endotoxin removal by direct hemoperfusion with an adsorbent column using polymyxin B-immobilized fiber ameliorates systemic circulatory disturbance in patients with septic shock. Am J Kidney Dis.

[CR53] Van Landeghem L, Mahe MM, Teusan R, Leger J, Guisle I, Houlgatte R (2009). Regulation of intestinal epithelial cells transcriptome by enteric glial cells: impact on intestinal epithelial barrier functions. BMC Genom.

[CR54] Vergnolle N, Cirillo C (2018). Neurons and glia in the enteric nervous system and epithelial barrier function. Physiology (bethesda).

[CR55] Vincent JL, Marshall JC, Namendys-Silva SA, Francois B, Martin-Loeches I, Lipman J (2014). Assessment of the worldwide burden of critical illness: the intensive care over nations (ICON) audit. Lancet Respir Med.

[CR56] Wang Y, Ouyang Y, Liu B, Ma X, Ding R (2018). Platelet activation and antiplatelet therapy in sepsis: a narrative review. Thromb Res.

[CR57] Wegrzyn G, Walborn A, Rondina M, Fareed J, Hoppensteadt D (2021). Biomarkers of platelet activation and their prognostic value in patients with sepsis-associated disseminated intravascular coagulopathy. Clin Appl Thromb Hemost.

[CR58] Yang Z, Wang K (2015). Glial fibrillary acidic protein: from intermediate filament assembly and gliosis to neurobiomarker. Trends Neurosci.

[CR59] Yu YB, Li YQ (2014). Enteric glial cells and their role in the intestinal epithelial barrier. World J Gastroenterol.

[CR60] Zarzycka B, Seijkens T, Nabuurs SB, Ritschel T, Grommes J, Soehnlein O (2015). Discovery of small molecule CD40-TRAF6 inhibitors. J Chem Inf Model.

[CR61] Zhang LN, Wang XH, Wu L, Huang L, Zhao CG, Peng QY (2016). Diagnostic and predictive levels of calcium-binding protein A8 and tumor necrosis factor receptor-associated factor 6 in sepsis-associated encephalopathy: a prospective observational study. Chin Med J.

[CR62] Zhou Q, Verne GN (2018). Intestinal hyperpermeability: a gateway to multi-organ failure?. J Clin Invest.

[CR63] Zhu Y, Fan Z, Wang R, Xie R, Guo H, Zhang M (2020). Single-cell analysis for glycogen localization and metabolism in cultured astrocytes. Cell Mol Neurobiol.

